# STR-based feature extraction and selection for genetic feature discovery in neurological disease genes

**DOI:** 10.1038/s41598-023-29376-4

**Published:** 2023-02-11

**Authors:** Jasbir Dhaliwal, John Wagner

**Affiliations:** 1 Faculty of Information Technology, Monash University, Clayton, VIC 3800 Australia; 2 PsychoGenics Inc., Paramus, New Jersey 07652 United States of America

**Keywords:** Biomarkers, Machine learning, Genetic markers, Gene expression

## Abstract

Gene expression, often determined by single nucleotide polymorphisms, short repeated sequences known as short tandem repeats (STRs), structural variants, and environmental factors, provides means for an organism to produce gene products necessary to live. Variation in expression levels, sometimes known as enrichment patterns, has been associated with disease progression. Thus, the STR enrichment patterns have recently gained interest as potential genetic markers for disease progression. However, to the best of our knowledge, we are unaware of any study that evaluates and explores STRs, particularly trinucleotide sequences, as machine learning features for classifying neurological disease genes for the purpose of discovering genetic features. Thus, in this paper, we proposed a new metric and a novel feature extraction and selection algorithm based on statistically significant STR-based features and their respective enrichment patterns to create a statistically significant feature set. The proposed new metric has shown that the neurological disease family genes have a non-random AA, AT, TA, TG, and TT enrichment pattern. This is an important result, as it supports prior research that has established that certain trinucleotides, such as AAT, ATA, ATT, TAT, and TTA, are favored during protein misfolding. In contrast, trinucleotides, such as TAA, TAG, and TGA, are favored during premature termination codon mutations as they are stop codons. This suggests that the metric has the potential to identify patterns that may be genetic features in a sample of neurological genes. Moreover, the practical performance and high prediction results of the statistically significant STR-based feature set indicate that variations in STR enrichment patterns can distinguish neurological disease genes. In conclusion, the proposed approach may have the potential to discover differential genetic features for other diseases.

## Introduction

The Human Genome Project helped catalyze the genetic revolution by enabling research into the genetic roots of human health and disease, advancing our understanding of the gene itself, and impacting numerous areas of research and medicine. Two areas of tremendous impact are whole genome sequencing and single nucleotide polymorphism (SNP) genotyping. Whole genome sequencing is a process for determining an individual’s entire genetic makeup, that is, the sequence of nucleotides, the main component of which are the bases cytosine, guanine, adenine or thymine (denoted by C, G, A, and T, respectively). This includes both coding (i.e., genes) and non-coding regions. In particular, whole genome sequencing yields the complete set of gene sequences in the genome, which are the biological blueprints for building the proteins that perform most of the work in the body.

SNP genotyping, by contrast, is a way of measuring genetic changes, or polymorphisms, of the nucleotides at specific genetic locations. Because SNPs provide a means for an organism to regulate the production of gene products necessary for life^1^ but are often disrupted as part of disease processes, patterns of SNPs can then be used to understand genetic variability in groups or cohorts of genes or genomes, including the variability in gene expression between individuals or tissues in healthy and diseased states.

Another genetic pattern involved in regulating gene expression levels and variability in healthy and diseased states is short tandem repeats (STRs). An STR is a section of short, repeated sequences of nucleotides, or DNA motifs. These “repeat units” (RUs) typically range from 1 to 6 nucleotides in length and are commonly repeated up to several dozen times in an STR^2^. Note that it is common for genes to contain multiple STRs, and even multiple STRs that share the same RUs. Finally, since STRs contain multiple copies of RUs, these STR sequences of RUs are often referred to as STR enrichment patterns. In addition to SNPs and STRs, other factors affecting gene expression include structural variants^3^ and environmental factors^4^.

Determining the SNP and STR enrichment patterns has recently gained interest as potential genetic markers or therapeutic targets for disease progression^5^. In earlier work^6^, we discovered SNP genetic features by proposing a novel feature extraction algorithm based on SNP enrichment patterns from large-scale, real-world biological datasets (all 49 human tissues from the Genotype-Tissue Expression portal) for tissue-specific gene prediction. Our results demonstrated that variation in the SNP enrichment patterns could distinguish the gene and the tissue in which the gene is expressed. Encouraged by these results, here we aim to discover statistically significant STR-based genetic features by proposing a novel STR-based feature extraction and selection algorithm for gene prediction. In particular, we have chosen to focus on trinucleotide repeat units as amino acids, which are the building blocks of protein, are also made up of a series of three nucleotides each. There are 64 possible combinations of three nucleotides, of which 61 represent amino acids and three are stop signals (codons). Trinucleotides have biological importance as they are involved in protein synthesis. For example, trinucleotides CCC and AAA code for proline and lysine amino acids, respectively. For more details on the genetic code, see reference^7^). Thus, it would be interesting to study how enrichment patterns obtained from trinucleotide repeats can be used to model machine learning features.

STRs were first discovered in 1989 when Litt and Luty^8^ noted the widespread distribution of dinucleotide TG repeat of length *n* in the human cardiac actin gene. More recently, abnormal STR enrichment patterns have been associated with diseases. Dai et al.^9^ showed that there is an increased frequency of dinucleotide GA repeats of lengths 13 to 16 (in SH2D2A gene) in multiple sclerosis patients relative to controls. Another study^10^ noted a shifted allelic frequency distribution between Alzheimer’s patients and controls for CpG-CA repeat lengths. Similarly, Myers^11^ reaffirmed that the trinucleotide sequence CAG is usually repeated about 20 times in the HTT gene but that an approximate doubling of repeats to 40 or more is necessary for the manifestation of Huntington’s disease. Several types of spinocerebellar ataxia (SCA) also involve repeat expansion: the normal range for SCA10 pentanucleotide ATTCT repeats is 12 to 25; for SCA12 trinucleotide CAG repeat is 9 to 24; and for SCA36 hexanucleotide GGCCTG repeat is 5 to 13^12^. (For excellent reviews on the current body of work around STRs, we refer the reader to references^13–16^.). These results indicate that healthy and disease genes are enriched with varying di- and tri-nucleotide repeat frequencies. Thus, the frequency patterns of STRs may be potential genetic markers. More recently, Zhang et al.^17^ analyzed dinucleotide frequency patterns of the whole-genome sequences from more than 1300 prokaryotic species. Their results show that the dinucleotides AC, AG, CA, CT, GA, GT, TC, and TG are well-conserved across various genomes in contrast to the frequencies of other dinucleotides that vary considerably among species. In a separate study, Liew et al.^18^ evaluated 22 coding features often used for DNA classification for three species, including trinucleotides ATG, TAA, TAG, and TGA, where the last three trinucleotides are stop codons. The researchers concluded that features that are efficient for yeast or C. elegans are generally inefficient for humans.

Prior research has shown that certain trinucleotides, such as ATA, ATT, and TAT, are favored in beta sheet secondary structures, while TTA is favored in alpha helix secondary structures. On the other hand, trinucleotide AAT is favored in beta bulges, which can be thought of as an irregularity that forms in a beta sheet when the regular hydrogen bonding of the beta sheet is disrupted^19^. In contrast, trinucleotides, such as TAA, TAG, and TGA, are stop codons that signal the termination of the current protein synthesis process. An initial exploration of our approach showed that the neurological disease family genes have a non-random AA, AT, TA, TG, and TT enrichment pattern. More specifically, we show that the mentioned trinucleotides are more enriched in neurological disease family genes than all human genes. Importantly, this result is consistent with prior work indicating that alpha helices are destroyed, and beta sheets are formed when a protein misfolds^20^. In addition, premature termination codon mutations in genes have been associated with some neurological diseases^21^. Given that these dinucleotide sequences are contained in the trinucleotide sequences favored in secondary structures and stop codons that play a role in protein misfolding and premature termination codon mutations, respectively, and by extension, some neurological diseases, we decided to analyze the frequency distribution of trinucleotide repeats in neurological disease family genes as these patterns may be potential genetic features.

Thus, in this paper, we evaluate and explore variations in trinucleotide repeat patterns as machine learning features for predicting neurological disease genes for the purpose of discovering genetic features. More specifically, in this paper, we raise several research questions, including (1) Are STRs more enriched in the neurological disease family genes than in all genes in the human genome?; (2) Are there significant statistical differences in STR enrichment patterns between the two classes of genes?; (3) What are the statistically significant repeat units?; (4) What are the non-random patterns indicated by statistically significant repeat units between the two classes of genes?; and (5) How can statistically significant repeat units be used to model machine learning features efficiently?. These questions are important as they progressively lead to machine learning modeling using STR-based features. In answering these questions, we generated a list of 426 genes that have known associations with neurological diseases. This list of genes is one of the scientific contributions of this work; links to this list and the list of the 16,863 human genes used are in the Availability of data and materials section. The contributions of this paper include:

- Proposition of a new metric that we call as repeat sum to analyze STR enrichment patterns as our study focuses on analyzing the frequency distribution of trinucleotide repeats in the entire gene sequence. Therefore, the longest repeat metric commonly used in most literature is not suitable for our study.
- Proposition of how three different (within- and between) normalization techniques can be adapted to analyze the repeat sum of repeat units. We also show that the results obtained from this metric support prior research on protein misfolding and premature termination codon mutations, which are involved in some neurological diseases.
- Identification of statistically significant repeat units at significance levels of $p<0.05$, $p<0.01$, and $p<0.001$, respectively. Selected repeat units are then used during the feature extraction and selection phase.
- Proposition of a novel feature extraction and selection algorithm based on statistically significant STR-based features and their respective enrichment patterns.
- A comprehensive analysis of our experimental results using our machine learning model with four different (within- and between) normalization techniques.

## Preliminary definitions and notation

This section sets the notation and defines the terms we will refer to throughout the paper. However, most of the relevant terminology for each section is described in more detail in that section itself.

Our work focuses exclusively on trinucleotide sequences. However, other repeated sequences such as mono-, di-, tetra-, penta-, and hexa-nucleotide repeats also exist. Another term that is often used synonymously with STR is repeat unit (RU). More specifically, we refer to each trinucleotide of an STR as an RU. Moreover, as an RU has three nucleotides, there are 64 ($4^3=64$) possible combinations. (Of note, an RU may be a potential STR-based genetic feature.) Following on, we refer to the number of times an RU is repeated as repeat count (RC). Summing the RCs results in a repeat sum (RS) for each RU. A RC can further be categorized as a maximum repeat count (max RC) (to represent the maximum number of repeats); minimum repeat count (min RC) (to represent the minimum number of repeats); or most common repeat count (most RC) (to represent the most common repeat). Finally, we refer to the expansion of an RU based on the RC as a repeat.

**Figure 1:**
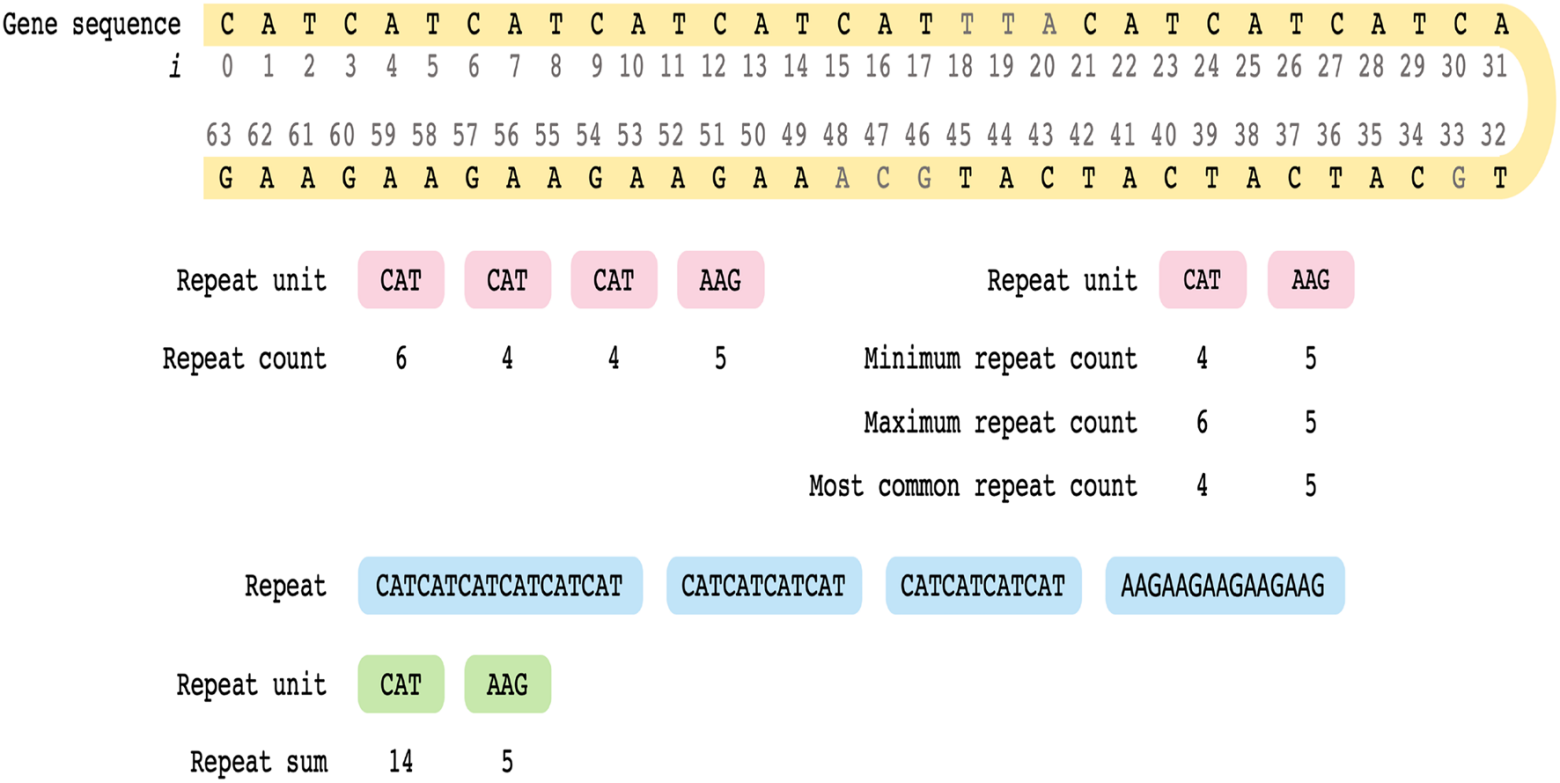
An example using gene sequence to demonstrate the general terminologies used throughout the paper.

We will walk through an example using a gene sequence to demonstrate the terminologies used and their relations in Fig. 1. The sequence comprises 64 nucleotides, and two RUs exist: CAT and AAG, as they are repeated more than three times. Throughout the literature, there are several cut-off points for the repeat count, and here, we are following the minimum threshold value suggested by Lai and Sun^22^ for humans, i.e., four. Moreover, the statistical analysis performed later on neurological and human genes indicated that nearly all the repeat units have a min RC of four. Thus, RU CAT at positions $i=0$, $i=21$ and $i=34$ have RCs of 6, 4 and 4, respectively. Furthermore, max RC is 6, min RC is 4, and most RC is 4. Combining RU CAT with the RCs gives us a repeat of length 18 (CATCATCATCATCATCAT) and two repeats of length 12 (CATCATCATCAT). Following on, RS for RU CAT is 14. We can conclude that this gene is more enriched with RU CAT (has high expression levels of CAT) but less enriched with RU AAG (has low expression levels of AAG).

## Methods

We present the statistical analysis and machine learning analysis sections. The first section answers the first three research questions, while the second answers the last two. We performed statistical analysis to understand the neurological genes’ characteristics to prepare the data and features for machine learning analysis.

### Statistical analysis

#### Data

We generated two datasets; the first dataset consists of 426 neurological disease family genes. We obtained these genes in a two-step process. First, we queried the 40 neurological disease families identified in^23^ as search terms in the Online Mendelian Inheritance in Man (OMIM) database. Then, we identified 49 neurological genes that are known to be caused by STR variation in recent review papers^15,24^. This resulted in 453 distinct genes downloaded from the National Center for Biotechnology Information (NCBI) database.

In contrast, the second dataset comprises the entire human genome and precisely 19,142 protein-coding genes downloaded from the NCBI. We further preprocessed these datasets by selecting genes with at least an RU (recall an RU is a trinucleotide of an STR) that is repeated more than three times (RC > 3). This resulted in 426 neurological disease family genes (of which 47 are neurological genes known to be caused by STR variation), which we call NEU and 16,863 human genes, which we call HUM. Links to these GRCh38 genes are provided in the Availability of data and materials section.

#### Descriptive statistics

We performed descriptive statistics on all 64 trinucleotide RUs and their respective RSs of neurological and human genes.

#### Inferential statistics

We converted RSs of RUs into informative measures of STR enrichment to discover STR-based genetic features. Furthermore, to test the efficacy of RS as a metric, we consider the following three factors:

- **Length** Length is a between-sample factor affecting the results as longer sequence genes generally have more STRs than shorter sequences. The pseudocode of the algorithm that normalizes by length is presented as Algorithm 1. First, pseudocode NRL makes a left-to-right pass over the gene sequence to store RU and RS in a dictionary, *D*. Then, another left-to-right pass is made over *D* to normalize the RS of each RU by length. Hereafter we refer to the described normalization as Len Norm.
- **Count** Counts of RS is a within-sample factor as it indicates how enriched an RU is compared to the other RUs within the gene itself. The pseudocode of the algorithm that normalizes by counts is presented as Algorithm 2. The main difference to Algorithm 1 is that we sum the counts of RS in dictionary *D* in Step 3 to be used for normalization in Step 5. Hereafter we refer to the described normalization as Cnt Norm.
- **Maximum–minimum count** Maximum-minimum counts of RS are also a within-sample factor. Therefore, the algorithm’s pseudocode that normalizes by maximum-minimum counts of RS is presented as Algorithm 3. The main difference to Algorithm 2 is that we compute the maximum and minimum counts of RS in *D* in Step 3 to compute the difference between them in Step 4. This difference is then used for normalization in Step 6. Thus, hereafter we refer to the described normalization as MM Norm.

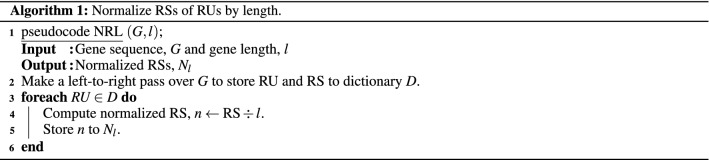
 
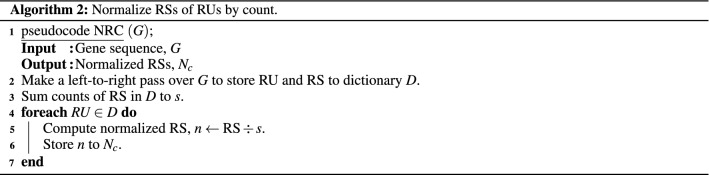
 
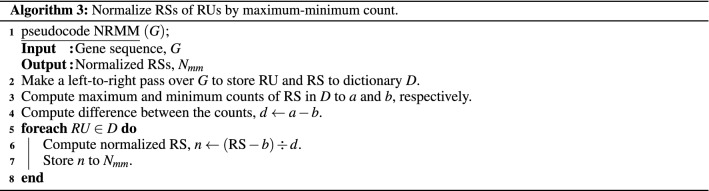


As RSs of the datasets exhibited a non-normal distribution, we chose a non-parametric test to run. Thus, having employed the described data normalization techniques, we implemented the Mann–Whitney U test in Python using the pingouin.mwu() function to test the null hypothesis of this study, i.e., there will be no statistically significant differences between the RSs of the two datasets. All analysis utilized a significance level of $p<0.05$, $p<0.01$ and $p<0.001$. Moreover, as 64 tests are conducted for all possible trinucleotide RUs, multiple hypothesis correction, particularly the two-stage step-up method of Benjamini et al.^25^ at the 0.1 family-wise error rate, is applied to identify statistically significant STR-based genetic features.

### Machine learning analysis

#### STR-based feature extraction and selection algorithm

Here, we propose the pseudocode based on statistically significant RUs and their respective enrichment patterns. More specifically, we call an RU most significant RU (MSRU) if significant differences exist. We obtained the MSRUs by sorting the RUs based on the lowest adjusted p-values. In contrast, we obtained the least significant RU (LSRU) by sorting the RUs based on the highest adjusted p-values while ensuring the MSRUs and LSRUs do not overlap.

At a high level, the algorithm extracts and selects MSRUs and their RCs to generate a statistically significant STR-based feature set based on MSRUs. The pseudocode MSFS, which we refer to as Algorithm 4, begins by making a left-to-right pass over a dataset, *G*. We assume *G* has been preprocessed to contain genes with at least an MSRU $\in$ MSRU-*k* that is repeated more than three times. MSRU-*k* refers to the top *k* statistically significant RUs. We only store an MSRU and RC to dictionary *D* if its RC is more than three. (Each MSRU holds a list of RCs).

Then, we create three empty lists: $L_{max}$, $L_{min}$, and $L_{com}$, to represent the max-, min-, and most RCs of each MSRU. We quantity the term “many”, “fewer”, and “common” STRs by storing the RCs in the above three lists. (Even though a gene may have several common STRs, our implementation code uses Python’s mode function (version 3.9.5) that returns the first encountered RC.).


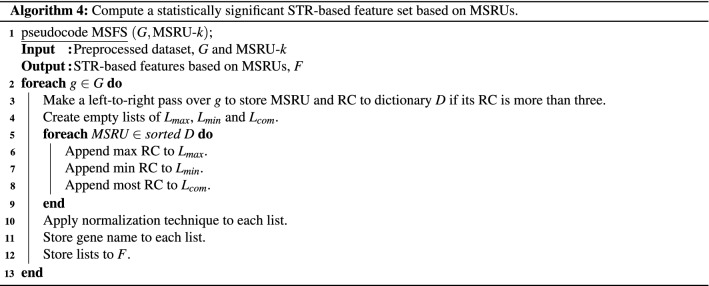


After storing RCs to their respective lists, we used the normalization techniques described in the Inferential statistics section on RCs instead of on RSs. Thus, for example, we employed Len Norm by dividing each RC with the gene length of Algorithm 1; Cnt Norm by dividing each RC with the summed counts of RS (of Steps 2 to 3 of Algorithm 2); and finally, MM Norm via the maximum and minimum counts of RS (of Steps 2 to 4 of Algorithm 3) on each RC. Furthermore, as RCs of MSRUs generally depict a bell curve, indicating a normal distribution, we chose to explore z-score normalization, which we will refer to as Zce Norm.

On a separate note, to create a non-statistically significant STR-based feature set based on LSRUs, we replace MSRU-*k* of Steps 3 and 5 of Algorithm 4 with LSRU-*k*.

#### Classification

The input to a machine learning classifier is the STR-based features extracted and selected from gene sequences, and the class labels are gene names. Here, we chose the support vector (SVM) as the classifier for two main reasons. The first is to test the features’ discriminating power obtained using our STR-based feature extraction and selection algorithm. This is because SVM is known to work relatively well on classes with clear separation boundaries^26^. Thus, if the discriminating power of our STR-based features is low (no apparent boundaries exist between genes), low prediction accuracy is expected. The second is to see if our machine learning model can run on many genes using a laptop, as SVM is also known to be computationally expensive for large datasets^26^.

We optimized the SVM classifier using the following hyperparameters: C [0.1, 0.5, 1, 10], gamma [0.1, 0.5, 1, 10], and kernel type [’linear’,’rbf’], via grid search stratified-3-fold cross-validation on 15% of NEU.

We designed two experiments to find the answer to the fifth research question. The first experiment aims to demonstrate the efficacy of employing MSRU-based features on the remaining 85% of NEU. Thus, we generated five test sets based on MSRU-*k* and LSRU-*k*, where $k \in [5, 10, 15, 20, 23]$. Note that the largest number for *k* is 23, as we are interested in all the RUs at significance level $p<0.01$. See Table 1 for details. For example, each gene in Test-1 contains at least an MSRU-5 and LSRU-5. This is so that we can evaluate the performance of the top *k* statistically significant features against the top *k* non-statistically significant features. We further evaluated the performance of these feature sets by comparing them against baseline features comprising all the 64 possible RUs. Finally, the second is another control experiment that illustrates the efficacy of our algorithm for gene prediction if a gene does not have any MSRUs. Thus, Test-6 contains a gene with at least an RU from all the 64 possible RUs, i.e., 85% of NEU.

Likewise, we repeated the above-described experiments on another dataset, i.e., HUM. This is a control dataset where we aim to evaluate the machine learning model’s efficacy on non-disease genes. However, as it is computationally impossible to run the entire human genes on the model and is not the focus of the paper, we chose a random sampling method commonly used by researchers to create a representative genome sample instead. To do so, we select a gene if the random number generated at that time was less than the threshold value of 1/6, resulting in a pool of genes we refer to as a random run. (While exploring several threshold values, we found that using threshold value 1/6 gave us a large sample of representative genes that may run on a laptop.) Moreover, we repeated the Mann–Whitney U test described in Inferential statistics section on each random run to see the statistical differences between their RUs. Thus, we ended up random sampling HUM for five random runs, as shown in Table 1. For example, RR = 1 represents the first random run while RR=2 represents the second random run. We repeated the experiments on each random run.

In addition, as an alternative disease dataset, we tested our approach on the delayed speech and language development genes with the Unified Medical Language System Concept Unique Identifier (UMLS CUI) of C0454644 identified from the DisGeNET database^27^. We then downloaded these genes from NCBI, resulting in 558 distinct genes. Similar to NEU and HUM datasets, we only used genes with at least an RU repeated more than three times, resulting in 535 genes, which we call DEL. Links to these GRCh38 genes are provided in the Availability of data and materials section.

**Table 1:** The test sets and the number of genes used for the experiments are presented. (NA indicates not applicable).

Dataset	RR	Test-1	Test-2	Test-3	Test-4	Test-5	Test-6
	*k*	5	10	15	20	23	64
NEU	NA	176	237	251	262	281	362
HUM	1	1063	1557	1667	1792	1949	2917
	2	1025	1515	1608	1738	1921	2857
	3	1055	1492	1593	1735	1886	2862
	4	1031	1458	1564	1698	1868	2820
	5	1055	1517	1617	1734	1886	2781
	MEAN	1046	1508	1610	1739	1902	2847
DEL	NA	183	349	417	425	431	535

#### Evaluation metrics

We evaluated our experiments using five machine learning evaluation metrics: F1-score, precision, recall, accuracy, and Matthews correlation coefficient (MCC). We report the macro-average scores for the first three metrics as we have a balanced dataset, i.e., each gene has the same number of samples, i.e., three per gene to represent the max-, min- and most RCs. Thus, the machine learning model was also evaluated using stratified-3-fold cross-validation. The stratified K-fold is a commonly accepted cross-validation technique. Stratification is a process that ensures each fold is a good representative of each class, which is dependent on the number of samples. This technique splits the dataset into groups known as folds and, in our case, three folds, where two folds are used for training the model while the remaining fold is used for testing the model. Finally, we evaluated the time-space complexity of our approach using total run time and peak RAM usage reported by the Python’s time.time() and resource.getrusage() functions.

## Results

### Analyze statistical results

#### Descriptive statistics

The summary of mean, maximum, minimum, and standard deviation of RSs, can be found as Supplementary Table S1 online. Based on the results, we conclude that generally, the neurological disease family genes have a larger mean of RSs than human genes. Moreover, variation in the standard deviations of RSs indicates that STRs enriched in a particular gene are less enriched in another gene.

**Figure 2:**
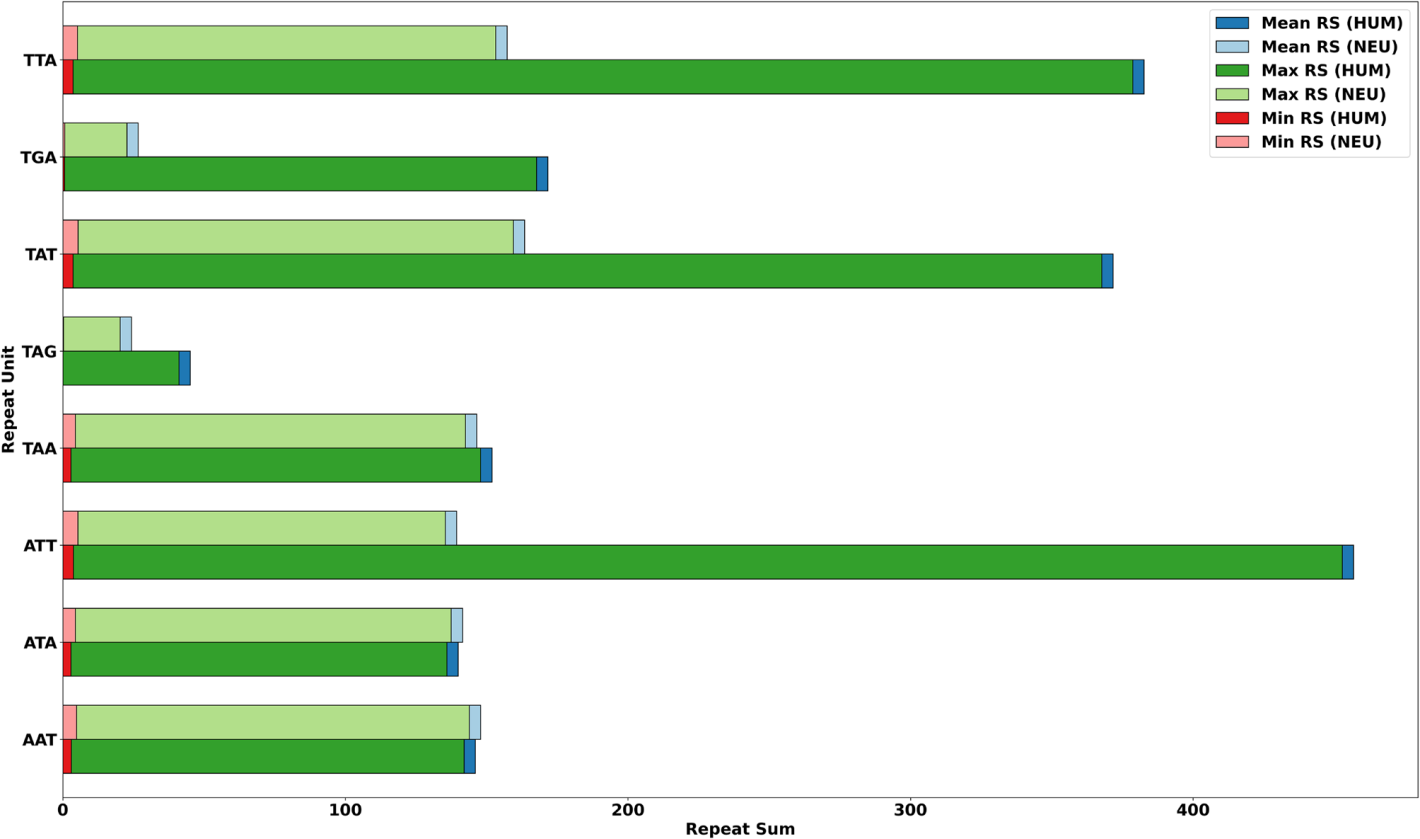
Mean-, max- and min- RS distribution for the RUs of interest.

We draw attention to RUs enriched with AA, AT, TA, TG, and TT as these RUs have considerably large maximum values. (These RUs are highlighted in Supplementary Table S1 online and visualized in Fig. 2.) Thus, in NEU, the maximum value for RUs AAT, ATA, ATT, TAA, TAT, and TTA are obtained from gene CSMD1 that is associated with autism, schizophrenia, and Parkinson disease late-onset^28^. Furthermore, the maximum value for RUs TAG and TGA are obtained from genes PHYH and TCF4, which are associated with retinitis pigmentosa and Pitt-Hopkins syndrome, respectively^28^. In contrast, in HUM, only the maximum values for RUs AAT and ATA are from gene CSMD1, while the rest of the values are obtained from different genes. The maximum values for RUs ATT, TAT, and TTA are obtained from gene ERC1, and the maximum value for RU TAA is obtained from gene CNTNAP2. Both these genes are associated with neurological diseases; (ERC1 with pontocerebellar hypoplasia type 2E and CNTNAP2 with Pitt–Hopkins-Like syndrome 1 and autism 15^28^). Likewise, the maximum value for RUs TAG and TGA are obtained from genes CTNND2 and SORBS2, which are also associated with neurological diseases, particularly familial adult myoclonic epilepsy and retinitis pigmentosa-23, respectively^28^. In other words, even though our results showed that HUM has higher maximum repeat sums than NEU for the following RUs: ATT, TAA, TAG, TAT, TGA, and TTA, the genes that contributed to the higher maximum repeat sums are neurological genes. However, these genes were not picked up via the applied selection process for the NEU dataset generation, and the HUM dataset refers to all human genes.

A protein’s function is determined mainly by its structure, which is a combination of primary, secondary, and tertiary structures. A protein’s primary structure is its sequence of amino acids. A protein’s secondary structure comprises regular substructures like alpha helices and beta sheets. These substructures and the unstructured pieces of the protein are then “folded up” yielding the protein’s full three-dimensional shape, the tertiary structure. Some diseases are associated with the destabilization of protein structure. One common feature of such diseases is the destabilization of alpha helices and the formation of beta sheets^20^. This is called protein misfolding. As the name suggests, this event causes proteins to be misfolded and later deposited in the body. Protein misfolding has been associated with some neurological diseases, including Alzheimer’s disease, Parkinson’s disease, and Huntington’s disease^20^.

Certain repeat units, such as ATA, ATT, and TAT, are favored in beta sheets, while TTA is favored in alpha helices. On the other hand, the repeat unit AAT is favored in beta bulges, which can be thought of as an irregularity that forms in a beta sheet when the regular hydrogen bonding of the beta sheet is disrupted^19^. In contrast, repeat units TAA, TAG, and TGA are stop codons that signal the termination of the current protein synthesis process. Premature termination codons occur from single nucleotide mutations that convert a triplet nucleotide codon into one of three stop codons (TAA, TAG, or TGA) and are involved in some neurological diseases^29^, including Alzheimer’s disease^21^. Thus, the proposed repeat sum metric shows that the neurological disease family genes have a non-random pattern of AA, AT, TA, TG, and TT enrichment. More specifically, we show that the mentioned trinucleotide repeat units are more enriched in neurological disease family genes than all human genes. This is an important result, as it supports prior research that has established that certain trinucleotides are favored during protein misfolding and premature termination codon mutations. More specifically, this result is consistent with prior work indicating that alpha helices are destroyed, and beta sheets are formed when a protein misfolds^20^, as well as stop codons being favored during premature termination codon mutations^29^. This suggests that the proposed metric has the potential to identify patterns that may be genetic features in a sample of neurological genes.

Besides protein misfolding and premature termination codon mutations, there are other mechanisms underlying many neurological diseases. This includes oxidative stress in Alzheimer’s disease, Parkinson’s disease, Huntington’s disease, progressive supranuclear pals; mitochondrial dysfunction in Alzheimer’s disease, Parkinson’s disease; fragmentation of neuronal Golgi apparatus in amyotrophic lateral sclerosis, corticobasal degeneration, Alzheimer’s disease, Parkinson’s disease, Creutzfeldt-Jakob disease, SCA2^30^; gene silencing in fragile × syndrome^31^; and splicing interference in autism^32^.

Therefore, given that the above dinucleotide sequences are contained in the trinucleotide sequences favored in secondary structures and stop codons that play a role in protein misfolding and premature termination mutations, respectively, and by extension, some neurological diseases, we decided to explore these patterns further.

#### Inferential statistics

**Table 2:**
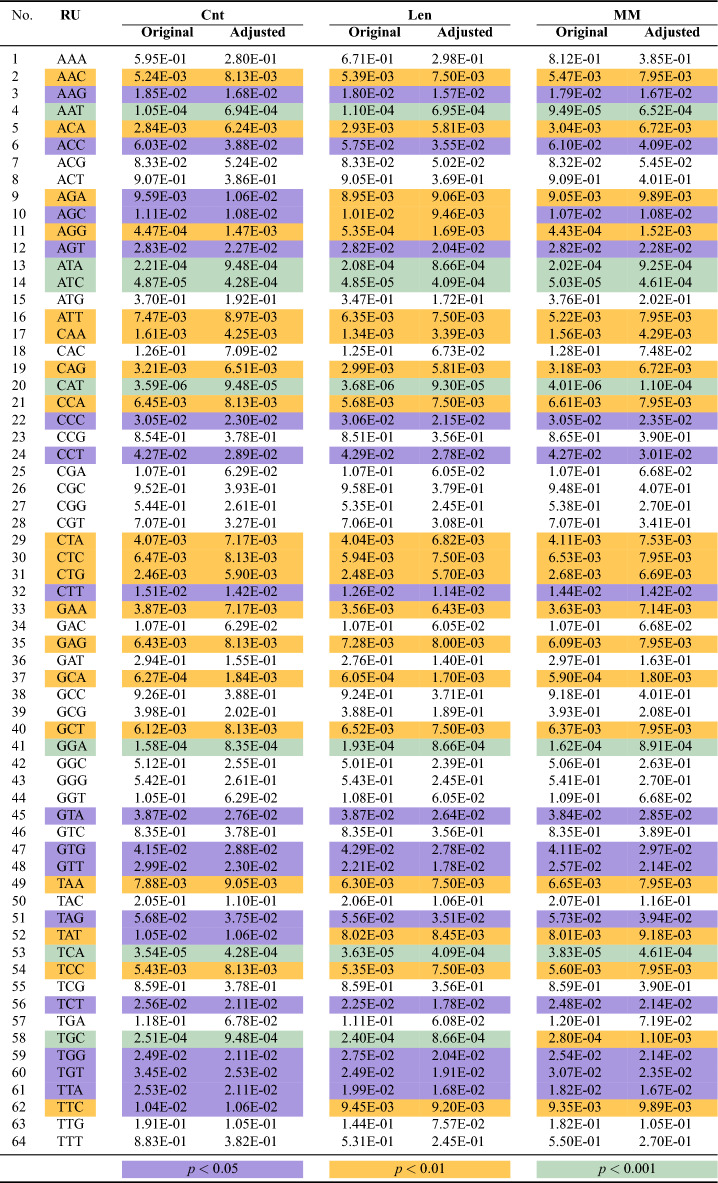
The RUs and their respective original and adjusted *p* values.

Using the two-stage step-up method of Benjamini et al.^25^, we identified forty-one RUs that are statistically significant at a family-wise error rate of 0.1 for all three techniques. More specifically, the original and adjusted *p* values of these features at significance levels of $p<0.05$, $p<0.01$, and $p<0.001$, respectively, are highlighted with three different colors in Table 2. All the RUs are ordered alphabetically. Note that, due to the slight difference in the adjusted *p* values, a slight difference in RUs order is seen in all three techniques. Thus, the Cnt Norm technique produced seven statistically significant RUs at $p<0.001$, sixteen at $p<0.01$, and eighteen at $p<0.05$. Likewise, the Len Norm produced seven statistically significant RUs at $p<0.001$, twenty at $p<0.01$, and fourteen at $p<0.05$. Finally, the MM Norm produced six statistically significant RUs at $p<0.001$, twenty at $p<0.01$, and fifteen at $p<0.05$.

**Figure 3:**
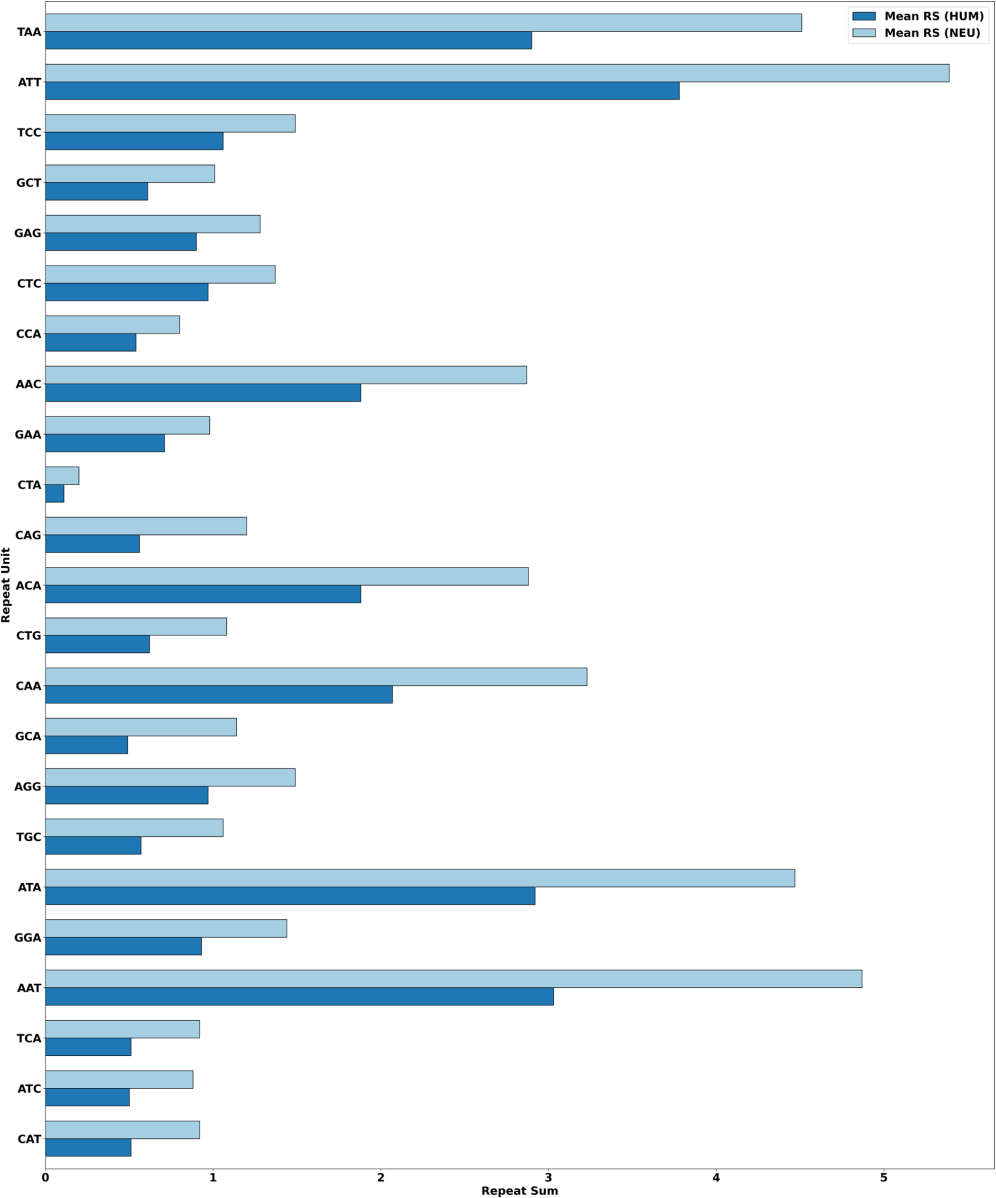
Mean distribution of RS of the most significant RUs (MSRUs) between the two datasets, where MSRU CAT has the lowest adjusted *p* value, followed by MSRU ATC, and so on.

**Figure 4:**
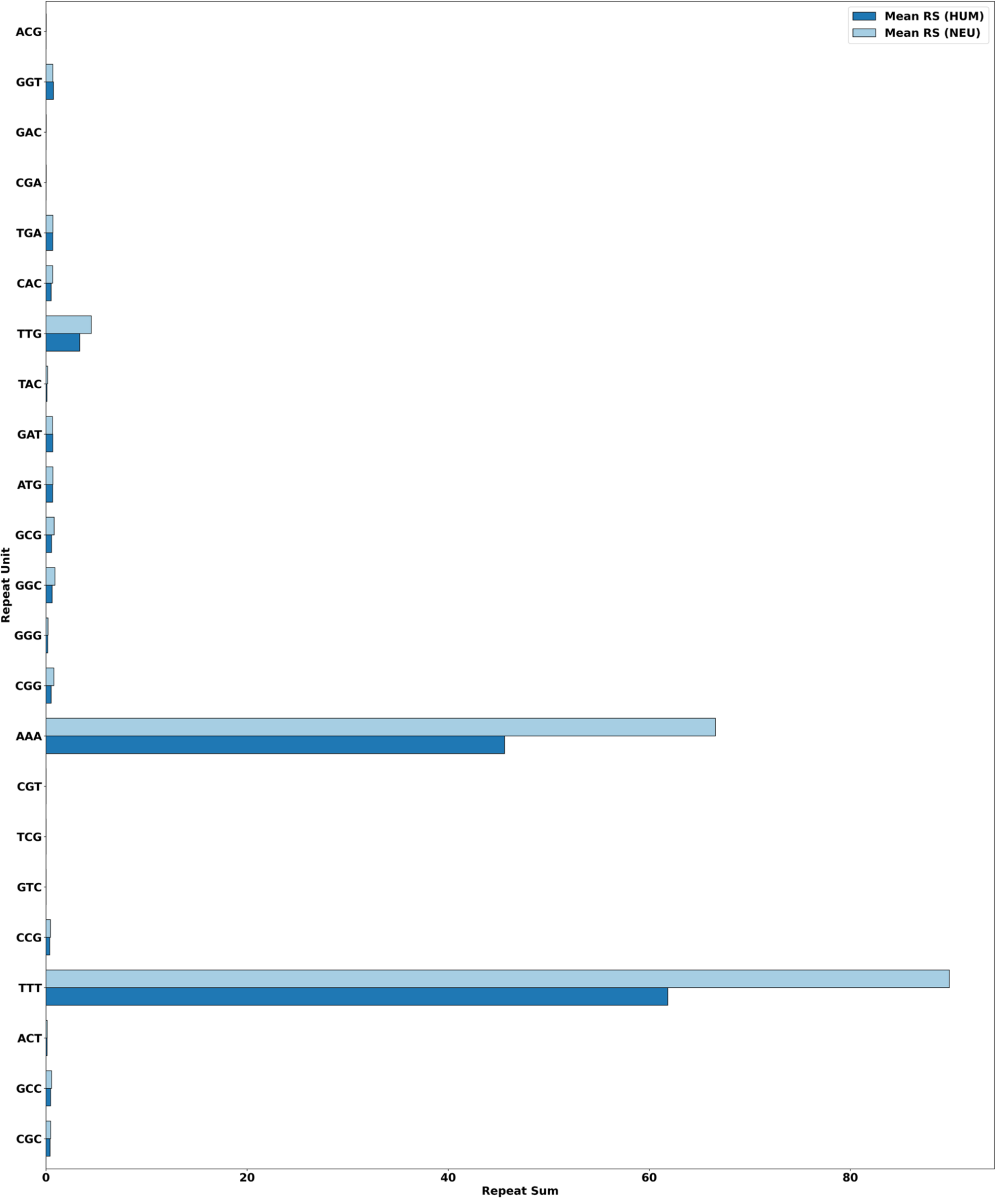
Mean distribution of RS of the least significant RUs (LSRUs) between the two datasets, where LSRU CGC has the highest adjusted *p* value, followed by LSRU GCC, and so on.

We expected the above results as the three normalization techniques are highly correlated, involving trinucleotide frequency counts or the number of nucleotides. However, we chose to adapt them as the paper focuses on analyzing the frequency distribution of trinucleotide repeats across neurological genes. In this paper, we chose to explore the RUs obtained from the Cnt Norm technique at significance levels $p<0.01$ and $p<0.001$, respectively. Recall that MSRUs are sorted based on the lowest adjusted *p* values, in contrast to LSRUs, which are sorted based on the highest values. For the mean distribution of RSs between neurological and all human genes for MSRUs and LSRUs, we refer the reader to Figs. 3 and 4. (In future work, we will explore the order of the MSRUs and LSRUs obtained from the remaining two normalization techniques).

### Analyze machine learning results

#### Visualization

**Figure 5:**
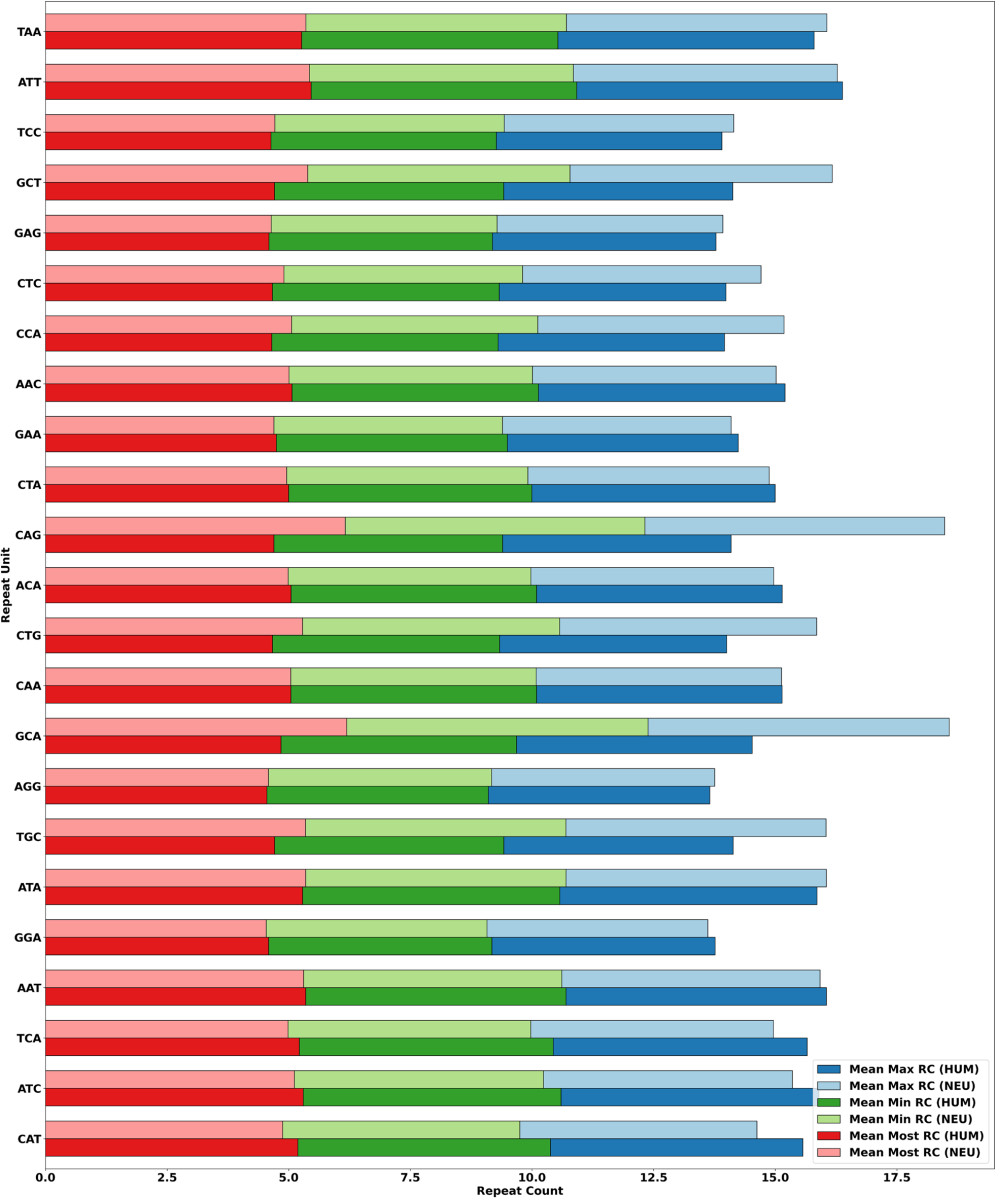
Mean distribution of max-, min-, and most- RC of the most significant RUs (MSRUs) between the two datasets, where MSRU CAT has the lowest adjusted *p* value, followed by MSRU ATC, and so on.

**Figure 6:**
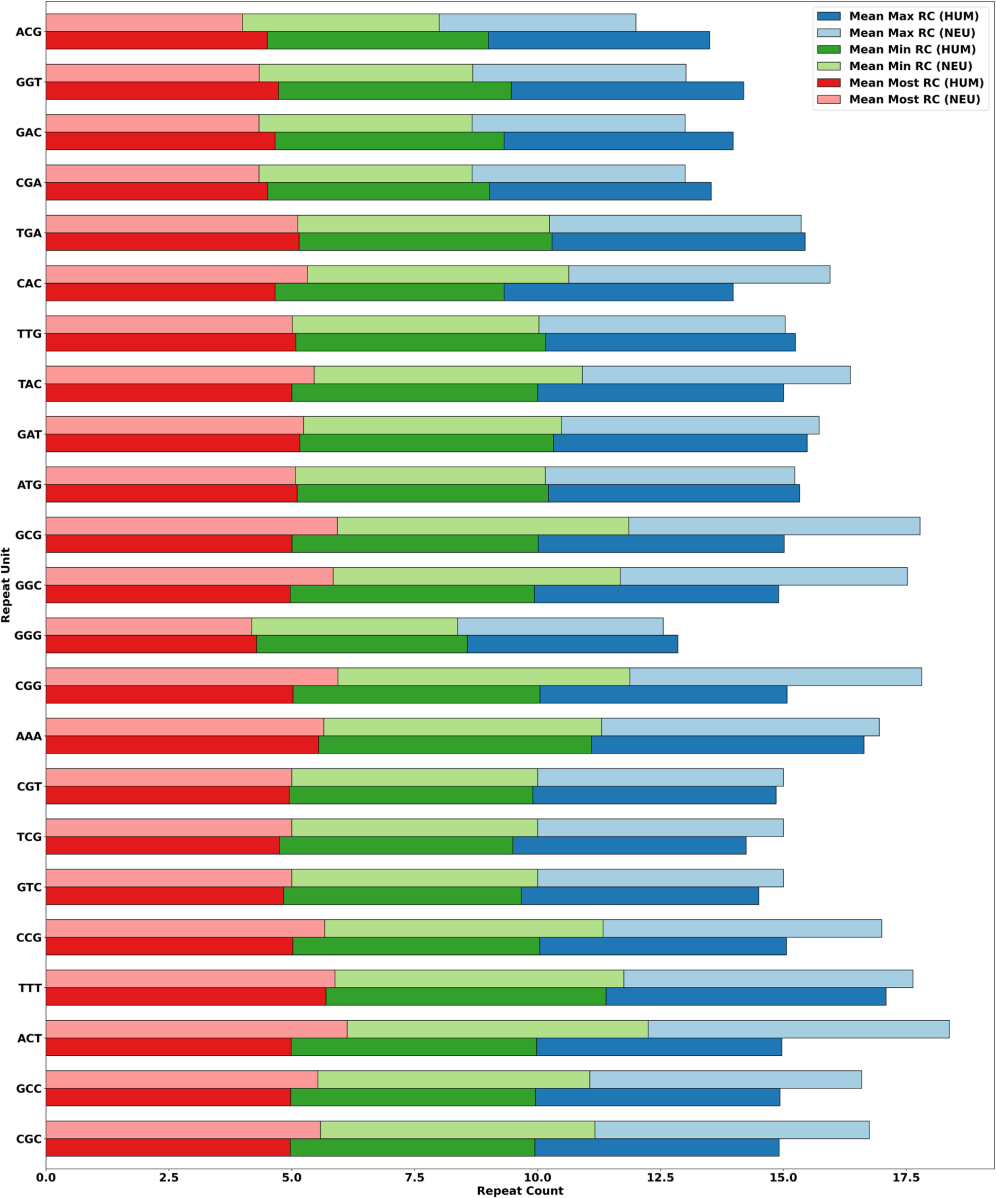
Mean distribution of max-, min-, and most- RC of the least significant RUs (LSRUs) between the two datasets, where LSRU CGC has the highest adjusted *p* value, followed by LSRU GCC, and so on.

This section addresses research question 4). Thus, we first demonstrate the max RC (representing more enriched STR), min RC (representing less enriched STR), and most RC (representing either more or less enriched STR) of MSRUs in Fig. 5 and LSRUs in Fig. 6, respectively. These figures contain the mean distribution of max-, min-, and most- RCs for MSRUs and LSRUs. The MSRUs are presented based on their lowest adjusted *p* values, while the LSRUs are presented based on their highest adjusted *p* values. For example, referring to Fig. 5, the order of MSRUs is as follows: the first one MSRU refers to MSRU-1, while all the twenty-three MSRUs refer to MSRU-23. In other words, MSRU CAT has the lowest adjusted *p* value while MSRU ATC has the second lowest adjusted *p* value, and so on.

The above results assert that RCs of MSRUs may have discriminating powers as STR-based machine learning features. (A good feature has a low degree of similarities with features of other genes).

#### Hyperparameter optimization

The optimal hyperparameters for SVM are as follows: C (0.1), gamma (0.1), and kernel (linear).

#### STR-based machine learning model

Experiments were conducted on datasets of NEU, HUM, and DEL datasets on an otherwise ideal 3.1 GHz Dual-Core Intel Core i5 with 16 GB main memory. The operating system was macOS Monterey.

**Table 3:** Effect of varying the top *k* features on NEU test sets. Accuracy indicated by Acc, F1-score indicated by F1, Precision indicated by Pre, and Recall indicated by Rec. Also, the time is reported in the format mm:ss while the peak memory usage is in Megabytes.

*k*	Model	Most significant RUs				Least significant RUs				All RUs			
		Acc	F1	Pre	Rec	Acc	F1	Pre	Rec	Acc	F1	Pre	Rec
5	SVM+ Len	0.74	0.68	0.65	0.74	0.09	0.07	0.07	0.09	0.90	0.88	0.86	0.90
	SVM+ Cnt	0.72	0.65	0.63	0.72	0.09	0.07	0.06	0.09	0.91	0.88	0.87	0.91
	SVM+ MM	0.76	0.71	0.69	0.76	0.13	0.12	0.11	0.13	0.92	0.90	0.89	0.92
	SVM+ Zsc	0.82	0.78	0.76	0.82	0.20	0.18	0.17	0.20	0.96	0.95	0.94	0.96
**Time**		01:31				01:44				01:40			
**Mem**		245				248				246			
10	SVM+ Len	0.85	0.81	0.79	0.85	0.13	0.11	0.10	0.13	0.90	0.88	0.87	0.90
	SVM+ Cnt	0.84	0.81	0.79	0.84	0.13	0.11	0.10	0.13	0.89	0.86	0.85	0.89
	SVM+ MM	0.88	0.85	0.84	0.88	0.19	0.18	0.18	0.19	0.95	0.93	0.92	0.95
	SVM+ Zsc	0.92	0.90	0.88	0.92	0.28	0.25	0.24	0.28	0.96	0.95	0.95	0.96
**Time**		01:41				01:44				01:49			
**Mem**		257				253				255			
15	SVM+ Len	0.90	0.87	0.86	0.90	0.19	0.16	0.15	0.19	0.91	0.88	0.87	0.91
	SVM+ Cnt	0.89	0.86	0.84	0.89	0.19	0.16	0.15	0.19	0.89	0.87	0.85	0.89
	SVM+ MM	0.92	0.90	0.89	0.92	0.30	0.28	0.27	0.30	0.95	0.93	0.93	0.95
	SVM+ Zsc	0.95	0.93	0.92	0.95	0.39	0.36	0.35	0.39	0.96	0.95	0.95	0.96
**Time**		01:42				01:48				01:53			
**Mem**		256				255				257			
20	SVM+ Len	0.90	0.88	0.86	0.90	0.28	0.24	0.23	0.28	0.90	0.88	0.87	0.90
	SVM+ Cnt	0.91	0.89	0.87	0.91	0.27	0.24	0.23	0.27	0.89	0.87	0.86	0.89
	SVM+ MM	0.94	0.92	0.92	0.94	0.41	0.39	0.38	0.41	0.95	0.94	0.93	0.95
	SVM+ Zsc	0.95	0.94	0.93	0.95	0.54	0.50	0.49	0.54	0.96	0.95	0.94	0.96
**Time**		01:44				01:51				01:55			
**Mem**		257				257				258			
23	SVM+ Len	0.90	0.88	0.86	0.90	0.32	0.28	0.26	0.32	0.90	0.88	0.86	0.90
	SVM+ Cnt	0.90	0.87	0.86	0.90	0.30	0.26	0.24	0.30	0.89	0.86	0.85	0.89
	SVM+ MM	0.94	0.92	0.92	0.94	0.47	0.45	0.44	0.47	0.95	0.93	0.92	0.95
	SVM+ Zsc	0.96	0.95	0.94	0.96	0.58	0.54	0.52	0.58	0.96	0.95	0.95	0.96
**Time**		01:46				01:56				02:00			
**Mem**		261				260				261			

Table 3 compares results of using MSRU-based features against all features on NEU test sets using the SVM enhanced with various normalization techniques. Note that we only report accuracy, F1, precision, and recall scores in the paper due to space constraints. The MCC score is reported in Supplementary Table S2 online. There is a difference in scores reported by each normalization technique. Generally, the normalization techniques reported the best scores in the following order: Zsc Norm, MM Norm, Len Norm, and Cnt Norm. We expected this result because genes may have RUs with similar counts, further reducing the model’s prediction accuracy enhanced with Cnt and MM Norms. Likewise, the accuracy of Len Norm decreases if genes have RUs with similar counts and lengths. On the other hand, the Zsc Norm takes advantage of normally distributed RCs and uses average and standard deviation measures. As a result, we can conclude that a normalization technique affects the machine learning model’s performance.

Thus, the SVM+ Zsc model obtained the best scores. Except for $k=5$, nearly similar prediction scores are obtained regardless of whether MSRU-based features or all 64 features are employed. For example, when the top $k=10$ features (Test-2) are used, an accuracy of 0.92 was reported (cf. accuracy of 0.96 on all features). Comparable F1, precision, recall, and MCC scores were seen for all test sets. Generally, the running time is $\sim$ 2 minutes, and peak memory is $\sim$ 260 MB. Thus, the average running time per model is $\sim$ 30 seconds. We also expected the peak memory usage to be similar. This is because we processed the genes on the fly and stored the features efficiently; more memory is only used if the number of genes increases.

On the other hand, the LSRU-based features obtained low accuracy, as shown in Table 3. For example, SVM+ Zsc obtained an accuracy of 0.58 using the top $k=23$ features (Test 5). However, a much higher accuracy of 0.96 is observed when all 64 features are employed. Thus, we can conclude that high prediction scores of statistically significant STR-based features via SVM indicate clear class boundaries between genes. In contrast, lower prediction scores of non-statistically significant STR-based features via SVM indicate blur class boundaries between genes.

**Table 4:** Effect of varying the top *k* features on HUM test sets. Accuracy indicated by Acc, F1-score indicated by F1, Precision indicated by Pre, and Recall indicated by Rec. Also, the time is reported in the format mm:ss while the peak memory usage is in Megabytes.

*k*	Model	Most significant RUs				Least significant RUs				All RUs			
		Acc	F1	Pre	Rec	Acc	F1	Pre	Rec	Acc	F1	Pre	Rec
5	SVM+ Len	0.74	0.68	0.65	0.74	0.09	0.06	0.05	0.09	0.83	0.80	0.78	0.83
	SVM+ Cnt	0.65	0.57	0.54	0.65	0.07	0.05	0.04	0.07	0.82	0.79	0.78	0.82
	SVM+ MM	0.62	0.54	0.51	0.62	0.08	0.07	0.06	0.08	0.90	0.87	0.86	0.90
	SVM+ Zsc	0.80	0.74	0.72	0.80	0.12	0.10	0.09	0.12	0.93	0.91	0.90	0.93
**Time**		08:48				08:44				09:13			
**Mem**		771				774				756			
10	SVM+ Len	0.82	0.78	0.76	0.82	0.10	0.08	0.07	0.10	0.84	0.81	0.80	0.84
	SVM+ Cnt	0.77	0.72	0.70	0.77	0.08	0.07	0.06	0.08	0.83	0.80	0.79	0.83
	SVM+ MM	0.79	0.74	0.71	0.79	0.11	0.10	0.09	0.11	0.91	0.89	0.88	0.91
	SVM+ Zsc	0.89	0.85	0.84	0.89	0.15	0.13	0.13	0.15	0.94	0.92	0.91	0.94
**Time**		13:47				14:27				14:42			
**Mem**		783				798				791			
15	SVM+ Len	0.87	0.83	0.82	0.87	0.14	0.12	0.11	0.14	0.84	0.82	0.80	0.84
	SVM+ Cnt	0.83	0.79	0.77	0.83	0.12	0.10	0.09	0.12	0.83	0.81	0.80	0.83
	SVM+ MM	0.85	0.81	0.79	0.85	0.17	0.15	0.14	0.17	0.92	0.90	0.89	0.92
	SVM+ Zsc	0.92	0.90	0.89	0.92	0.23	0.21	0.20	0.23	0.94	0.93	0.92	0.94
**Time**		16:32				16:59				16:53			
**Mem**		821				808				825			
20	SVM+ Len	0.89	0.87	0.85	0.89	0.20	0.17	0.16	0.20	0.84	0.82	0.80	0.84
	SVM+ Cnt	0.87	0.83	0.82	0.87	0.19	0.16	0.15	0.19	0.84	0.81	0.80	0.84
	SVM+ MM	0.89	0.86	0.84	0.89	0.26	0.24	0.23	0.26	0.92	0.90	0.89	0.92
	SVM+ Zsc	0.94	0.92	0.91	0.94	0.34	0.32	0.30	0.34	0.94	0.93	0.92	0.94
**Time**		24:54				23:19				24:59			
**Mem**		930				939				847			
23	SVM+ Len	0.88	0.85	0.84	0.88	0.22	0.19	0.18	0.22	0.82	0.80	0.78	0.82
	SVM+ Cnt	0.86	0.82	0.81	0.86	0.20	0.17	0.17	0.20	0.82	0.79	0.78	0.82
	SVM+ MM	0.89	0.85	0.84	0.89	0.29	0.26	0.25	0.29	0.91	0.89	0.88	0.91
	SVM+ Zsc	0.93	0.91	0.90	0.93	0.38	0.35	0.33	0.38	0.94	0.92	0.91	0.94
**Time**		24:42				25:10				24:33			
**Mem**		915				909				941			

Likewise, in Table 4, we show the same measures for the control test sets, i.e., HUM test sets. We report the mean accuracy, F1, precision, recall, and MCC scores, as most have a standard deviation of 0.00 or 0.01. The MCC score is reported in Supplementary Table S3 online. Generally, the SVM+ Zsc model obtained the best scores. Again, except for $k=5$, nearly similar results were also observed for MSRU-based features and all 64 features. For example, when the top $k=10$ features (Test-2) are used, an accuracy of 0.89 was reported (cf. accuracy of 0.94 with all features). Furthermore, comparable scores were observed for all test sets. On the other hand, low accuracy, F1, precision, recall, and MCC scores were reported for LSRU-based features.

To further validate our approach, we repeated the feature extraction and selection steps on another disease dataset, i.e., DEL test sets. Like NEU, we show the top twenty-three statistically and non-statistically repeat units, selected via the Cnt Norm technique in Table 5. In addition, we note that NEU’s MSRUs have a significance level of $p<0.01$ in contrast to the DEL’s MSRUs that have a significance level of $p<0.000001$. Moreover, RU CAT, the first MSRU in NEU, is the last LSRU in DEL. (For the original and adjusted *p* values obtained for all the RUs via the three normalization techniques, see Supplementary Table S4 online.) We expected this result as different datasets will have different statistically significant RUs that may be potential genetic features. (In other words, generally, different datasets will have different repeat units).

**Table 5:** Comparison between statistically and non-statistically significant RUs of NEU and DEL datasets.

No.	Most significant RUs		Least significant RUs	
	NEU	DEL	NEU	DEL
1	CAT	CCC	CGC	CGT
2	ATC	ACC	GCC	AAA
3	TCA	CCA	ACT	TCG
4	AAT	GGC	TTT	GTC
5	GGA	GCG	CCG	TAC
6	ATA	AGG	GTC	TTT
7	TGC	TTG	TCG	ACT
8	AGG	CCG	CGT	CTA
9	GCA	TGT	AAA	TAG
10	CAA	GCC	CGG	TCT
11	CTG	TGC	GGG	CGA
12	ACA	GTT	GGC	CTT
13	CAG	CGG	GCG	CTC
14	CTA	TAT	ATG	GTA
15	GAA	TAA	GAT	CCT
16	AAC	TGA	TAC	TCC
17	CCA	ATT	TTG	GGG
18	CTC	GAG	CAC	TTC
19	GAG	GAT	TGA	ATC
20	GCT	AGC	CGA	GAC
21	TCC	ATA	GAC	CAA
22	ATT	CGC	GGT	ACA
23	TAA	GCT	ACG	CAT

However, unlike the NEU and HUM test sets, nearly similar results were observed from $k=5$ onwards for MSRU-based features and all 64 features. (See Table 6 for details.) For example, when the top $k=5$ features (Test-1) are used, an accuracy of 0.96 was reported (cf. accuracy of 0.95 with all features). Likewise, comparable scores were observed for all test sets where SVM+ Zsc model reported the best scores. (Supplementary Table S5 online contains the MCC score.) Thus, based on the experimental results, we can conclude that statistically significant STR-based features have almost the same discriminatory prediction power as all the STR-based features but are far better than non-statistically significant STR-based features while using little running time and memory.

**Table 6:** Effect of varying the top *k* features on DEL test sets. Accuracy indicated by Acc, F1-score indicated by F1, Precision indicated by Pre, and Recall indicated by Rec. Also, the time is reported in the format mm:ss while the peak memory usage is in Megabytes.

*k*	Model	Most significant RUs				Least significant RUs				All RUs			
		Acc	F1	Pre	Rec	Acc	F1	Pre	Rec	Acc	F1	Pre	Rec
5	SVM+ Len	0.93	0.91	0.90	0.93	0.06	0.04	0.03	0.06	0.90	0.87	0.86	0.90
	SVM+ Cnt	0.90	0.87	0.85	0.90	0.06	0.04	0.04	0.06	0.91	0.89	0.88	0.91
	SVM+ MM	0.91	0.88	0.87	0.91	0.09	0.08	0.07	0.09	0.92	0.90	0.90	0.92
	SVM+ Zsc	0.96	0.95	0.94	0.96	0.15	0.12	0.11	0.15	0.95	0.94	0.93	0.95
**Time**		02:12				02:08				02:09			
**Mem**		280				279				279			
10	SVM+ Len	0.82	0.78	0.76	0.82	0.08	0.06	0.05	0.08	0.88	0.86	0.85	0.88
	SVM+ Cnt	0.81	0.77	0.75	0.81	0.08	0.06	0.06	0.08	0.88	0.86	0.85	0.88
	SVM+ MM	0.85	0.81	0.80	0.85	0.14	0.13	0.12	0.14	0.93	0.91	0.90	0.93
	SVM+ Zsc	0.91	0.89	0.88	0.91	0.18	0.15	0.15	0.18	0.95	0.94	0.93	0.95
**Time**		03:00				02:53				03:01			
**Mem**		333				332				335			
15	SVM+ Len	0.82	0.78	0.77	0.82	0.11	0.09	0.09	0.11	0.88	0.85	0.84	0.88
	SVM+ Cnt	0.80	0.76	0.74	0.80	0.12	0.10	0.09	0.12	0.88	0.85	0.84	0.88
	SVM+ MM	0.86	0.82	0.81	0.86	0.23	0.21	0.20	0.23	0.93	0.91	0.90	0.93
	SVM+ Zsc	0.90	0.87	0.86	0.90	0.28	0.25	0.24	0.28	0.95	0.94	0.94	0.95
**Time**		03:14				03:12				03:18			
**Mem**		333				382				381			
20	SVM+ Len	0.86	0.83	0.81	0.86	0.19	0.16	0.15	0.19	0.89	0.86	0.85	0.89
	SVM+ Cnt	0.85	0.82	0.80	0.85	0.17	0.15	0.14	0.17	0.88	0.86	0.85	0.88
	SVM+ MM	0.89	0.87	0.86	0.89	0.33	0.31	0.30	0.33	0.93	0.92	0.91	0.93
	SVM+ Zsc	0.91	0.89	0.88	0.91	0.42	0.38	0.36	0.42	0.95	0.94	0.94	0.95
**Time**		03:13				03:14				03:16			
**Mem**		381				383				329			
23	SVM+ Len	0.87	0.83	0.82	0.87	0.25	0.22	0.21	0.25	0.88	0.86	0.85	0.88
	SVM+ Cnt	0.86	0.82	0.81	0.86	0.24	0.22	0.21	0.24	0.88	0.85	0.84	0.88
	SVM+ MM	0.90	0.87	0.86	0.90	0.43	0.40	0.39	0.43	0.93	0.91	0.91	0.93
	SVM+ Zsc	0.91	0.89	0.88	0.91	0.52	0.48	0.46	0.52	0.96	0.95	0.94	0.96
**Time**		03:17				03:18				03:23			
**Mem**		325				382				330			

Finally, to demonstrate the viability of our approach when a gene does not have any MSRUs, we tested our model with all 64 features. Table 7 summarizes the accuracy, F1, precision, and recall scores on the Test 6 of NEU, HUM, and DEL, respectively. The MCC score is reported in Supplementary Table S6 online. We observed an accuracy of 0.95 on 362 NEU genes, 0.83 on $\sim$ 2,847 HUM genes, and 0.94 on 535 DEL genes. Again, we report the mean scores for human genes as they only have a standard deviation of 0.01. On a separate note, running each random run on the model is a challenge in itself. This is because each run consists of a considerably large number of genes where conventionally, such numbers require a dedicated, high-performance cluster, and here, we are using a laptop. Hence, we can conclude that, generally, little running time and peak memory usage are used as the reported numbers are for all four models. Moreover, even with many genes, we still see the power of normalization techniques where SVM+ Zsc achieved the best scores.

**Table 7:** A separate experiment demonstrates the efficacy of our approach if a gene does not have any MSRUs on Test-6 of all three datasets. Accuracy indicated by Acc, F1-score indicated by F1, Precision indicated by Pre, and Recall indicated by Rec. Also, the time is reported in the format hh:mm:ss while the peak memory usage is in Megabytes.

*k*	Model	NEU				HUM				DEL			
		Acc	F1	Pre	Rec	Acc	F1	Pre	Rec	Acc	F1	Pre	Rec
64	SVM+ Len	0.84	0.81	0.80	0.84	0.74	0.70	0.68	0.74	0.83	0.80	0.79	0.83
	SVM+ Cnt	0.81	0.78	0.77	0.81	0.66	0.63	0.62	0.66	0.79	0.77	0.76	0.79
	SVM+ MM	0.94	0.93	0.92	0.94	0.81	0.78	0.77	0.81	0.92	0.89	0.88	0.92
	SVM+ Zsc	0.95	0.94	0.94	0.95	0.83	0.80	0.79	0.83	0.94	0.93	0.92	0.94
**Time**		01:55				1:24:42				03:17			
**Mem**		271				1084				401			

## Discussion

The purpose of our study is to analyze the frequency distribution of trinucleotide repeats in neurological genes, as these patterns may be potential genetic features. Thus, we evaluated and explored variations in trinucleotide repeat patterns as machine learning features for predicting neurological disease family genes. In doing so, we proposed a new repeat sum metric that indicated a non-random AA, AT, TA, TG, and TT enrichment pattern, where we show that the mentioned trinucleotides are more enriched in neurological disease family genes than all human genes. This is an important result, as it supports prior research that has established that certain trinucleotides, such as AAT, ATA, ATT, TAT, and TTA, are favored during protein misfolding. In contrast, trinucleotides, such as TAA, TAG, and TGA, are favored during premature termination codon mutations, as they are stop codons. In other words, our results are consistent with prior work that established protein misfolding and premature termination codon mutations as mechanisms involved in some neurological diseases, including Alzheimer’s disease. This further suggests that the proposed metric has the potential to identify patterns that may be genetic features in a sample of neurological genes.

Once we have identified the statistically significant repeat units, we proceeded to develop a novel STR-based feature extraction and selection algorithm. (These repeat units are potential genetic features.) In particular, we used the maximum repeat count (representing more enriched STR), minimum repeat count (representing less enriched STR), and most common repeat count (representing more or less enriched STR) of the repeat sum of each STR-based feature. The high prediction accuracy, low memory usage, and short running times, even on a large sample of genes using a laptop, indicated the efficacy of our approach. We also showed that the statistically significant STR-based features have similar discriminatory power as all 64 STR-based features but are better than the non-statistically significant STR-based features. Note that overfitting may have occurred in smaller test sets. However, a similar observation in prediction scores is seen even when the test set size increases. (Also, as observed in neurological as well as delayed speech and language development genes, different repeat units are selected at different significance levels. We expected this result as, generally, each disease has different genetic features.) Moreover, our approach can run a considerably large number of human genes using a laptop even though, conventionally, such numbers require a dedicated, high-performance cluster.

A limitation of the results is that our approach was run on the reference genome genes. The reference genome sequence is made up of several anonymous individuals, where approximately 70% of the sequence comes from a single individual^33^. Thus, it is highly unlikely that all known neurological diseases are present in the individuals that make up the reference genome. Also, interindividual variability of repeat size, an important biological factor for STRs, is not considered. (In other words, certain STRs have varying numbers of repeats among individuals.) Therefore, the machine learning model possibly encodes STR ‘fingerprints’ of genes rather than recapitulating biology. Nevertheless, the reference genome genes helped to fulfill our initial purpose of analyzing the frequency distribution of trinucleotide repeats in neurological genes to identify potential genetic features. In future work, based on the promising results obtained from this study, we intend to sequence genes of interest from neurological patients and test the efficacy of our machine learning model on the interindividual variability of repeat size.

Furthermore, a limitation of our approach is that the prediction accuracy will be reduced if the RUs have similar counts density, i.e., similar counts (including similar maximum and minimum counts), or even similar gene lengths. In such cases, the normalization technique that uses the average and standard deviation, i.e., z-score normalization, is a better alternative to normalization via counts, maximum-minimum, or even gene lengths. (This is demonstrated by the experimental results that show that the z-score normalization works best with our model.) Another limitation of our approach is that it currently works well on trinucleotide STRs; for example, a RU of three nucleotides can only have $4^3=64$ possible combinations. Therefore, it was sufficient for us to employ a naive algorithm to find all the trinucleotide STRs in a gene. However, finding hexanucleotide STRs using a naive algorithm will increase the running time of our program. This is because a hexanucleotide RU will have $4^6=4096$ possible combinations. There is a broad literature on finding STRs efficiently, and it is a separate research area. Thus, in future work, we will also explore the various STR detection software^34,35^.

As we were interested in analyzing all the STR-based genetic features in neurological disease genes, we used the entire gene sequences as inputs to the feature extraction and selection algorithm. However, depending on the purpose of the analysis, researchers may benefit by applying our algorithm to a wide range of problems, including the longest common substring in a string problem. For example, consider the problem of analyzing all the STR-based genetic features in the longest common substring of a gene sequence. In that case, the input to our feature extraction and selection algorithm will be the longest common substring of each gene instead of the entire gene sequence. We expect the accuracy to be almost similar regardless of the normalization technique employed. This is because the probability of the repeat units having similar counts density will be low as we are dealing with variable lengths of longest common substrings. Also, it is worth noting that the repeat count is a tunable parameter whose value depends on the disease or non-disease genes being studied and the purpose of analysis.

Another application of our approach is finding genetic features between diseases. Therefore, future work includes applying our STR-based algorithm to a wider range of disease families. We also anticipate extending our work to di- and hexa-nucleotide repeats. Various normalization techniques will be explored with the machine learning model as these repeats have a different counts density than trinucleotide repeats.

## Conclusions

The proposed new metric has shown that the neurological disease family genes have a non-random AA, AT, TA, TG, and TT enrichment pattern. More specifically, we show that the mentioned trinucleotides are more enriched in neurological disease family genes than all human genes. This is an important result, as it supports prior research that has established that certain trinucleotide repeat units, such as AAT, ATA, ATT, TAT, and TTA, are favored during the protein misfolding mechanism. This mechanism is involved in some neurological diseases, including Alzheimer’s disease and Parkinson’s disease. In contrast, trinucleotide repeat units, such as TAA, TAG, and TGA, are favored during premature termination codon mutations and are also involved in some neurological diseases, including Alzheimer’s disease. This suggests that the proposed metric has the potential to identify patterns that may be genetic features in a sample of neurological genes. Moreover, the practical performance and high prediction results of the statistically significant STR-based feature set indicate that variations in STR enrichment patterns can distinguish neurological disease genes. This is a conclusion drawn from a computer science perspective based on the results obtained from neurological, delayed speech and language development as well as random runs of human genes. This further suggests that our approach may have the potential to discover differential genetic features for other diseases.

In this paper, we have (1) introduced a new metric that we call as repeat sum for STR enrichment patterns analysis; (2) shown how three different (within- and between) normalization techniques can be adapted to analyze the repeat sum of repeat units; (3) identified the top 7, 16 and 18 statistically significant repeat units at significance levels of $p<0.001$, $p<0.01$, and $p<0.05$, respectively. These repeat units may be potential genetic features; (4) developed a novel feature extraction and selection algorithm based on statistically significant STR-based features and their respective enrichment patterns; and (5) provided a comprehensive analysis of our experimental results using our machine learning model with four different (within- and between) normalization techniques.

## Supplementary Information

Supplementary Information.

## Data Availability

The genes in datasets generated during the current study are available from the NCBI database. The NCBI links for the datasets are below: NEU—https://www.ncbi.nlm.nih.gov/datasets/tables/genes/?table_type=genes&key=b0f3dfee413e0952fa27b5faa00214f3, HUM—https://www.ncbi.nlm.nih.gov/datasets/tables/genes/?table_type=genes&key=9910463ff7ff2cbfccbdb17dbfdc7811, DEL— https://www.ncbi.nlm.nih.gov/datasets/tables/genes/?table_type=genes&key=dba8ffad2c6f0a5f95dd05d62e129c75.
